# Discovery of *Staphylococcus aureus* Adhesion Inhibitors by Automated Imaging and Their Characterization in a Mouse Model of Persistent Nasal Colonization

**DOI:** 10.3390/microorganisms9030631

**Published:** 2021-03-18

**Authors:** Liliane Maria Fernandes de Oliveira, Marina Steindorff, Murthy N. Darisipudi, Daniel M. Mrochen, Patricia Trübe, Barbara M. Bröker, Mark Brönstrup, Werner Tegge, Silva Holtfreter

**Affiliations:** 1 Institute of Immunology and Transfusion Medicine, Department of Immunology, University Medicine Greifswald, 17475 Greifswald, Germany; liliane.fernandes@med.uni-greifswald.de (L.M.F.d.O.); venkata.darisipudi@med.uni-greifswald.de (M.N.D.); daniel.mrochen@inp-greifswald.de (D.M.M.); patricia.truebe@gmail.com (P.T.); broeker@uni-greifswald.de (B.M.B.); 2 Helmholtz Centre for Infection Research, Department of Chemical Biology, 38124 Braunschweig, GermanyMark.Broenstrup@helmholtz-hzi.de (M.B.)

**Keywords:** *Staphylococcus aureus*, colonization, mouse, JSNZ, aurintricarboxylic acid, ATA, adhesion inhibitor, mupirocin, nose

## Abstract

Due to increasing mupirocin resistance, alternatives for *Staphylococcus aureus* nasal decolonization are urgently needed. Adhesion inhibitors are promising new preventive agents that may be less prone to induce resistance, as they do not interfere with the viability of *S. aureus* and therefore exert less selection pressure. We identified promising adhesion inhibitors by screening a library of 4208 compounds for their capacity to inhibit *S. aureus* adhesion to A-549 epithelial cells in vitro in a novel automated, imaging-based assay. The assay quantified DAPI-stained nuclei of the host cell; attached bacteria were stained with an anti-teichoic acid antibody. The most promising candidate, aurintricarboxylic acid (ATA), was evaluated in a novel persistent *S. aureus* nasal colonization model using a mouse-adapted *S. aureus* strain. Colonized mice were treated intranasally over 7 days with ATA using a wide dose range (0.5–10%). Mupirocin completely eliminated the bacteria from the nose within three days of treatment. In contrast, even high concentrations of ATA failed to eradicate the bacteria. To conclude, our imaging-based assay and the persistent colonization model provide excellent tools to identify and validate new drug candidates against *S. aureus* nasal colonization. However, our first tested candidate ATA failed to induce *S. aureus* decolonization.

## 1. Introduction

Nasal colonization with *Staphylococcus aureus* is a major risk factor for invasive staphylococcal infections [1,2]. In particular, infections caused by methicillin-resistant *S. aureus* (MRSA) have limited treatment options and are associated with higher morbidity and mortality [3]. To prevent endogenous infection as well as transmission within the hospital, newly admitted patients are routinely screened for MRSA colonization and decolonized using the antibiotic mupirocin [4]. However, increasing bacterial resistance to mupirocin with a prevalence exceeding 13% for MRSA [5] and restrictions for its use have created a need for alternatives [6,7]. In the past, several alternative interventions for the clearance of *S. aureus* nasal carriage have been explored, including antibiotics, such as neomycin [8], polysporin [9], and bacitracin [10], bacteriocins such as lysostaphin [11], as well as fatty acid derivatives (lauric acid monoesters) [12], and cationic synthetic polymers [13]. However, most of these candidates have failed to completely eradicate *S. aureus* nasal colonization. Hence, new approaches to combat *S. aureus* colonization are urgently required.

The first step towards an effective colonization of *S. aureus* in the nares and on other sites of the human body is the attachment of the bacteria to human epithelial cells. This process is facilitated by multifunctional and redundant adhesins on the staphylococcal surface that bind to host cell molecules. An up-to-now underexplored possibility to prevent or eliminate *S. aureus* colonization is to specifically interfere with this bacterial adhesion. Adhesion inhibitors that do not interfere with the viability of the bacteria are promising new preventive agents, because they exert less selective pressure and therefore do not foster the development of resistances [14,15]. Initial studies have been carried out in this context, but so far, they did not lead to clinically useful compounds [16].

The identification of adhesion inhibitors from large compound libraries requires high throughput screening approaches. In former investigations on the adhesion of *S. aureus* to epithelial cells, adherent bacteria were quantified by visual inspection with a microscope and counting [17] or by employing radioactively-labeled bacteria [18]. Alternatively, the adherent bacteria were quantified by determining colony-forming unit (CFU) values after their detachment from eukaryotic cells [19]. These approaches are impractical for the screening of hundreds and thousands of compounds, and they do not detect morphologic effects of the test compounds on the eukaryotic binding partner. Recently, a procedure was reported that is amenable to higher throughput. It utilizes an ELISA to measure binding of *S. aureus* to the major eukaryotic interaction partners fibronectin, keratin, and fibrinogen [20]. However, influences of the compounds on the phenotype of the eukaryotic cells cannot be detected in this screening procedure either.

The in vivo validation of candidate adhesion inhibitors requires a robust and sustained *S. aureus* colonization model. We have previously established a persistent murine nasal colonization model using the mouse-adapted *S. aureus* strain JSNZ [21,22]. Due to its long-term adaptation to the murine host, this strain is capable of inducing persistent colonization of the murine nose and gastrointestinal tract without the need for prior antibiotic treatment [23]. This model enables researchers for the first time to study host–pathogen interaction during persistent colonization in the mouse and also to evaluate decolonization drugs.

Here we report on the development and application of a high throughput microtiter plate-based phenotypic in vitro assay that quantifies the adhesion of *S. aureus* to human epithelial cells. The procedure utilizes fluorescence labeling of eukaryotic cell nuclei and bacteria after their adhesion, followed by detection with an automated microscope with image analysis, in combination with a pipetting robot for the distribution of substance libraries and for liquid handling. We performed a medium throughput screen of more than 4000 compounds, and subsequently characterized aurintricarboxylic acid (ATA) that was identified as the most potent adhesion inhibitor in this assay. The ability of ATA to eradicate nasal *S. aureus* colonization was assessed in our persistent *S. aureus* colonization model. While the standard-of-care antibiotic mupirocin completely eliminated *S. aureus* colonization within three days of treatment, ATA did not show an anti-bacterial effect in vivo. Nevertheless, our novel microscopy-based screening approach and the mouse-adapted strain JSNZ are powerful tools to identify and validate new drug candidates against *S. aureus* nasal colonization.

## 2. Materials and Methods

### 2.1. Epithelial Cells

For the screening procedure, the human epithelial lung cell line A-549 (ACC 107) was obtained from the German Collection of Microorganisms and Cell Cultures (DSMZ, Braunschweig, Germany). For the verification of the activity of hit substances, Human Nasal Epithelial Primary Cells (HNEPC) from Provitro GmbH (Berlin, Germany) were used. Both cell lines were cultivated under conditions recommended by their respective depositors. Cell culture reagents came from Invitrogen (Carlsbad, CA, USA) and Provitro GmbH (Berlin, Germany).

### 2.2. S. aureus Strains

For the screening procedures, the *S. aureus* strain N315 (ST5-CC5-MRSA) was used [24], kindly provided by Prof. Dr. K. Becker, University of Greifswald, Germany. For the development of the assay conditions *S. aureus* SA113 [25] (ST8-CC8-Methicillin-sensitive *S. aureus* (MSSA)) and its adhesion-deficient deletion mutant Delta tagO were kindly provided by Prof. Dr. A. Peschel, University of Tübingen, Germany [26]. For hit validation, the wild type *S. aureus* strains 50,307,270 (rifampicin resistant), 50,128,509 (MRSA), and 50,046,981 (MSSA) were kindly provided by the Institute for Microbiology, Immunology and Hospital Hygiene from the city hospital of Braunschweig, Germany. For the in vivo experiments, the mouse-adapted *S. aureus* strain JSNZ (ST88-CC88-MSSA) was employed [21].

### 2.3. Compounds

Six substance collections were investigated (Table 1). Aurintricarboxylic acid (ATA) was initially part of the LOPAC collection. For further testing, it was obtained from Sigma-Aldrich/Merck (Darmstadt, Germany) as the free acid.

### 2.4. Mice

Female C57BL/6NRj mice with Specific and Opportunistic Pathogen Free status (SOPF, *S. aureus*-free, 9 weeks old) were purchased from Janvier Labs (Saint-Berthevin, France). Females were selected because they are less prone to develop genital abscesses upon JSNZ colonization than males [21]. Moreover, males tend to be more aggressive and to present fight wounds that might get infected with *S. aureus*. After delivery, the animals were acclimatized for 7 days before starting the experiments. The animals were kept in individually ventilated cages (IVC, 4 animals/cage) under SOPF conditions with litter material. Food and water (acidified with HCl) were provided ad libitum.

### 2.5. In Vitro Adhesion Assay

A-549 cells were resuspended in RPMI 1640 containing 10% FCS and seeded at a density of 1 × 10^4^ cells per well in 100 µL medium into 96 well, black, optical bottom microtiter plates (sterile, cell culture-treated; order no. 165,305, Nalgene Nunc, Rochester, NY, USA). After an incubation period of 4 to 5 days under cell culture conditions at 37 °C, when the cells had formed a uniform confluent layer at the bottom of the wells, the incubation medium was removed and replaced by 75 µL infection medium (RPMI 1640 containing 1% FCS and 20 mM Hepes, pH 7.4). Test compounds were added with a pipetting robot equipped with a pin tool at a final concentration of 20 µM (pipetting robot: Evolution P3, PerkinElmer, Waltham, MA, USA; pin tool: FP3CB, 96 floating tube pins, 0.787 mm diameter, length: 33 mm, transfer volume 80 nL, V&P Scientific, Inc., San Diego, CA, USA). In each microtiter plate, four wells each of the following controls were included: Cell culture medium containing DMSO in concentrations corresponding to the substance testing and as positive control, 50 µg/mL polyinosinic acid (Sigma-Aldrich, Darmstadt, Germany), which has been shown before to reduce the adhesion of *S. aureus* to epithelial cells by approximately 50% at this concentration [29].

25 µL of *S. aureus* N315 suspension that had been grown overnight in Brain Heart Infusion (BHI) medium (Sigma-Aldrich, Darmstadt, Germany), washed twice with phosphate-buffered saline (PBS), and re-suspended in infection medium to an optical density (OD) OD_600_ = 1.0 (1 × 10^8^ CFU/mL), was added to each well, resulting in an assay concentration of OD_600_ = 0.25 (2.5 × 10^6^ CFU/well). After an incubation period of 1 h at room temperature (RT), non-adherent bacteria were removed by carefully washing the cell layer three times with PBS. The A-549 cells with adherent bacteria were fixed with 4% paraformaldehyde in PBS for 20 min at RT and afterwards washed twice with PBS. For the detection of adherent bacteria 50 µL/well of a primary rabbit antibody against *S. aureus* lipoteichoic acid (Acris Antibodies GmbH, Herford, Germany) diluted 1:5000 in PBS with 1% bovine serum albumin (BSA) was added. After 35 min at RT, 50 µL of 4′,6 diamidino-2-phenylindol-dihydrochloride (DAPI-solution, Sigma-Aldrich, Darmstadt, Germany) for the detection of A-549 cell nuclei was added at a final concentration of 1 µg/mL. For some preliminary experiments in the course of evaluation, the cytoplasm was stained with CellTracker^TM^ Red CMTPX (Molecular Probes^®^, Invitrogen, Carlsbad, CA, USA) by incubating with a final concentration of 5 µM together with DAPI. After 10 min at RT and three washing steps with PBS, 50 µL/well of the secondary mouse antibody anti-rabbit Alexa Fluor^®^ 488 (Invitrogen) was added at a dilution of 1:1000 in PBS/1% BSA. After an incubation time of 45 min and washing with PBS, bacteria and A-549 cells were analyzed with the automated microscope ImageXpress Micro (IXM, Molecular Devices, Sunnyvale, CA, USA) and the dedicated software MetaXpress. The initial screening of the substance collections was carried out in duplicate in two independent experiments. In the reevaluation of the initial hits, different concentrations of active compounds were used (5, 10, 20, 50, 100 µM, for ATA also lower concentrations).

For the determination of the effect of ATA on precolonized cells, the adhesion assay was modified: After the initial 1 h incubation of the A-549 cells with bacteria without addition of compounds, the wells were washed carefully three times with PBS. Fresh infection medium and ATA were added to the cells at concentrations of 0.95 and 2.2 µg/mL. After further incubations for 1, 2, 3, 4, and 5 h, the cells were washed three times with PBS, followed by the microscope-based quantification procedure. For each time point controls without ATA were carried out, providing the reference values.

### 2.6. Quantification of Epithelial Cells and Bacteria

Each plate was imaged with the automated microscope. For each well, nine images from different sites with a size of 0.4 × 0.4 mm were acquired. A 20× objective and the fluorescence filters “DAPI” (377 and 447 nm for excitation and emission, respectively) for the detection of A-549 nuclei and “FITC” (475 and 536 nm for excitation and emission, respectively) for the quantification of Alexa Fluor^®^ 488 labeled bacteria were used. For some preliminary experiments in the course of evaluation, stained cytoplasm was visualized with the fluorescence filter “Texas Red” (560 and 624 nm for excitation and emission, respectively). Each image was analyzed with the MetaXpress software modul “Transfluor” and the mode “Vesicle area per cell”. With this mode, the bacterial area per DAPI stained cell nucleus was quantified. The average was calculated from the nine different images per well. Performance of the assay was evaluated in 92 wells without added compounds; 4 wells contained 50 µg/mL polyinosinic acid as positive control. In addition, the signal from the adherent bacteria (Alexa Fluor^®^ 488 fluorescence; excitation/emission at 485 nm/535 nm) was determined with a fluorescence microtiter plate reader (Fusion Universal Microplate Analyser, PerkinElmer, Waltham, MA, USA).

### 2.7. Cytotoxicity Assay

The cytotoxicity of our hit compounds on A-549 cells was quantified using a 3-(4,5-dimethylthiazol-2-yl)-2,5-diphenyltetrazolium bromide (MTT) assay following the procedure by Mosmann [30], modified by Sasse [31]. Briefly, sub-confluent A-549 cells were washed with PBS without Ca^2+^ and Mg^2+^, trypsinized, and resuspended in DMEM containing 10% FCS. 60 µL of serial dilutions of the test compounds were added to 120 µL aliquots of a cell suspension (5000 cells) in 96 well microtiter plates in duplicate. Blank and solvent controls were incubated under identical conditions. After 24 h, 20 µL MTT in PBS was added to a final concentration of 0.5 mg/mL. After 2 h, the precipitate of formazan crystals was centrifuged and the supernatant was discarded. The precipitate was washed with 100 µL PBS and dissolved in 100 µL 2-propanol containing 0.4% hydrochloric acid. The microplates were gently shaken for 20 min to ensure a complete dissolution of the formazan, and finally the absorption was measured at 595 nm using an ELISA plate reader. The percentage of viable cells was calculated and the mean was determined with respect to the controls with medium only.

### 2.8. Investigation of the Antimicrobial Activity

Aliquots of 120 µL of an overnight culture of *S. aureus* N315 in BHI medium were washed, adjusted to OD_600_ = 0.015, corresponding to approximately 5 × 10^5^ CFU/mL, and added to 60 µL of a serial dilution of the test compounds in BHI. After an incubation time of 18 h at 37 °C without shaking under moist conditions, the OD_600_ was measured with a microtiter plate reader (Fusion Universal Microplate Analyser, PerkinElmer, Waltham, MA, USA). The lowest concentration that completely suppressed growth defined the MIC values.

### 2.9. Preparation of the S. aureus Inoculum for In Vivo Experiments

*S. aureus* JSNZ strain was grown over night in BHI medium at 37 °C and 200 rpm. Thereafter, the culture was diluted to OD_595_ = 0.05 in BHI medium and cultivated at 37 °C and 200 rpm until the mid-logarithmic phase (OD_595_ = 2.0–2.5). Cells were harvested by centrifugation for 10 min at 8000× *g*, resuspended in BHI containing 14% sterile glycerine, and frozen at −80 °C. Before intranasal inoculation, bacteria stocks were thawed on ice, washed once with 40 mL PBS, and reconstituted in PBS to the desired concentration (1 × 10^10^ CFU/mL) based on the optical density of the suspension (OD_595_ = 1; equals 2.6 × 10^8^ CFU/mL). The actual bacterial dose was determined by plating serial dilutions of the inoculum on LB agar plates right after intranasal colonization. Plates were incubated over night at 37 °C and CFU were counted the following day.

### 2.10. Preparation of Substances for Intranasal Application

For initial experiments, ATA was dissolved in three different carrier substances for intranasal application: Poloxamer 407, Softisan 649/Vaseline 9:1, and PBS. Poloxamer 407 powder (Sigma-Aldrich, Darmstadt, Germany) was dissolved in sterile distilled water to the desired concentration and homogenized over night by gently shaking at 4 °C. This substance was stored at 4–8 °C and maintained on ice before intranasal inoculation. Softisan 649/Vaseline 9:1 (*w*/*w*) (Fagron GmbH & Co. KG, Barsbüttel, Germany), hereafter referred to as S/V, was warmed up to 65–80 °C for 10–30 min to obtain a liquefied solution suitable for pipetting. Since mupirocin 2% (Turixin^®^, GlaxoSmithKline GmbH & Co, München, Germany) was also formulated with S/V, this drug was also liquefied by warming to 80 °C for 15 min to enable pipetting of small volumes. ATA (Sigma-Aldrich, Darmstadt, Germany) was dissolved in sterile distilled water to 10–20%, thereafter diluted to the required concentration with Poloxamer (20% *w*/*v*) or with liquefied S/V.

### 2.11. Treatment of Colonized Mice with Drug Carriers, ATA and Mupirocin

To exclude a direct effect of the drug carrier itself on the bacterial load, we determined the impact of the three drug carriers PBS, 20% Poloxamer 407, and Softisan 649/Vaseline 9:1 (*w*/*w*) in a persistent *S. aureus* nasal colonization model. Mice were colonized intranasally with 0.7–1.0 × 10^8^ CFU *S. aureus* JSNZ (5 µL per nostril) under mild isoflurane anesthesia. Starting on day 3 after colonization, mice were treated once daily for 7 consecutive days with 10 µl of the respective carriers or left untreated. All substances were applied into the nasal cavity using a Hamilton^©^ syringe (100 µL Microliter Syringe, 22s-gauge, Hamilton, Bonaduz, Switzerland) with a shortened and blunted needle. The blunted needle was inserted a few millimeters into the anterior nares to ensure application of the substances within the nasal cavity. Gastrointestinal colonization was examined via stool samples that were collected from individual mice in septic cages on day 0, 3, 6, and 10 after colonization. Mice were weighed and visually inspected for any symptoms of infection on a daily basis. On day 6 and 10 after colonization, mice were sacrificed by isoflurane overdose. The nose including the nasal cavities as well as the cecum were collected for evaluation of the bacterial load.

To determine the anti-adhesive capacity of ATA, mice were colonized with JSNZ for three days as described above and afterwards treated once per day for consecutive 7 days with ATA or left untreated. Five microliters of ATA were slowly applied in each nostril (10 µL in total) using the blunted Hamilton^©^ syringe. The compound was applied at different concentrations (0.5%, 2%, 5%, or 10%) in two different carriers. ATA-Poloxamer was cooled on ice while ATA-S/V was warmed to 65–80 °C to turn liquid before intranasal application. The reference substance mupirocin was liquefied at 65–80 °C and applied in the clinically used dose of 2.0% (5 µL/nostril) following the same procedure. Both ATA and mupirocin were not affected by heating in this temperature range as verified by LC-MS controls and mupirocin sensitivity testing, respectively (data not shown) [8]. Animals were sacrificed by isoflurane overdose after 0, 3, or 7 days of treatment, and nose and cecum were obtained as detailed above.

### 2.12. Determination of the Bacterial Load

Stool samples were adjusted to 0.2 g/mL with sterile PBS followed by homogenization for 20 min at 1400 rpm and 4 °C using a Thermo Mixer C shaker (Eppendorf, Hamburg, Germany). The nose and cecum were weighed, transferred to autoclaved homogenizer tubes containing zirconium oxide beads (diameter: 1.4/2.8 mm, Precellys, France), and filled up with 1 mL sterile PBS. Cecum samples were homogenized at 6000 rpm for 2 × 20 s with a 15 s interval. Noses were homogenized at 6500 rpm for 2 × 30 s with a 5 min interval. Homogenized samples were serially diluted; 10 µL of each dilution were plated out in triplicate on *S. aureus* Chromagar plates (CHROmagar, France) and enumerated the next day.

### 2.13. Ethics Statement

Animal experiments received ethical approval from the responsible State authorities (Landesamt für Landwirtschaft, Lebensmittelsicherheit und Fischerei Mecklenburg-Vorpommern, 7221.3-1-018/19). The experiments were performed in accordance with the German Animal Welfare Act (Deutsches Tierschutzgesetz), the EU Directive 2010/63/EU for animal experiments and the Federation of Laboratory Animal Science Associations (FELASA). All animal experiments comply with the ARRIVE guidelines.

### 2.14. Statistics

Data analysis was performed using the GraphPadPrism6 package (GraphPad Software, Inc., La Jolla, California, USA). Group-wise comparisons were conducted using the Mann-Whitney test or Welch’s unpaired *t*-test, as indicated in the particular graph. Paired samples (i.e., disease activity scores) were compared using the Friedman test and Dunn’s multiple comparison test for post hoc analyses.

## 3. Results

### 3.1. Development of a Screening Method for the Identification of S. aureus Adhesion Inhibitors

Our first aim was the development of an automated microscopy-based method that allows the screening of several thousand substances for compounds that inhibit the adhesion of *S. aureus* to human lung epithelial cells (A-549) [17,32,33,34]. Since the bacteria were found to adhere to the plastic surface of the microtiter plates (not shown), it was important to grow the A-549 cells to a dense confluent layer before performing the adhesion assay. Initial experiments showed a linear correlation between the amount of bacteria used and the bacterial adhesion to A-549 cells (Figure S1). For the subsequent screening campaigns we found an intermediate amount (OD_600_ = 0.25, corresponding to 2.5 × 10^7^ CFU/mL) to be optimal for the performance of the assay. An incubation time of one hour at RT was found most suitable, since longer incubation times or higher temperatures led to a bias due to bacterial proliferation and/or compounds that have an influence on bacterial growth rather than on adhesion. For the reliable detection and quantification of the bacteria in the automated microscope, fluorescence labeling was employed, and several methods were evaluated, including pre-incubation of the bacteria with fluorescein isothiocyanate (FITC) and Syto 9. The most suitable approach was paraformaldehyde fixation at the end of the incubation period, followed by the staining of adherent bacteria with a commercial primary antibody against *S. aureus* lipoteichoic acid and an Alexa Fluor 488-conjugated secondary antibody, together with DAPI staining of the eukaryotic cell nuclei.

During assay development, we investigated the adhesion of *S. aureus* N315 to A-549 cells in relation to the growth phase. *S. aureus* was cultivated for 2 h, 4 h, 6 h, 8 h, 19 h, and 24 h in BHI, harvested, washed, and resuspended in infection medium. The adhesion of overnight grown bacteria was comparable to the adhesion of mid-log phase bacteria (Figure S2). For technical reasons we preferred to use overnight cultures in our approach.

The adherence was measured with an automated microscope by a quantitative detection of the ‘vesicle area per cell’, the “vesicles” representing the Alexa Fluor-labeled bacteria and the “cells” the DAPI-stained nuclei of the A-549 lung epithelia. In parallel, adherent bacteria were quantified by scanning the plates with a fluorescence microtiter plate reader. The microscopic approach led to more reliable and reproducible values than the data obtained with the microtiter plate reader. The reliability of both assays was assessed by performing the adhesion test in 92 wells without addition of compounds, including controls (Figure S3). For the microscopy-based test, the Z’ factor was determined to be 0.54, which was considered to be of sufficient quality for the screening procedure [35], whereas the Z’ factor for the microtiter plate-based test was only 0.12 and considered insufficient.

The functionality of the assay in terms of the detection of influences on adhesion was validated with the *S. aureus* wild type strain SA113 and its isogenic mutant SA113 Delta tagO, whose efficiency of adhesion was reduced by approximately 50% as shown before [34]. Different ratios of bacteria to A-549 cells showed the expected differences between the strains (Figure S1).

**Figure 1:**
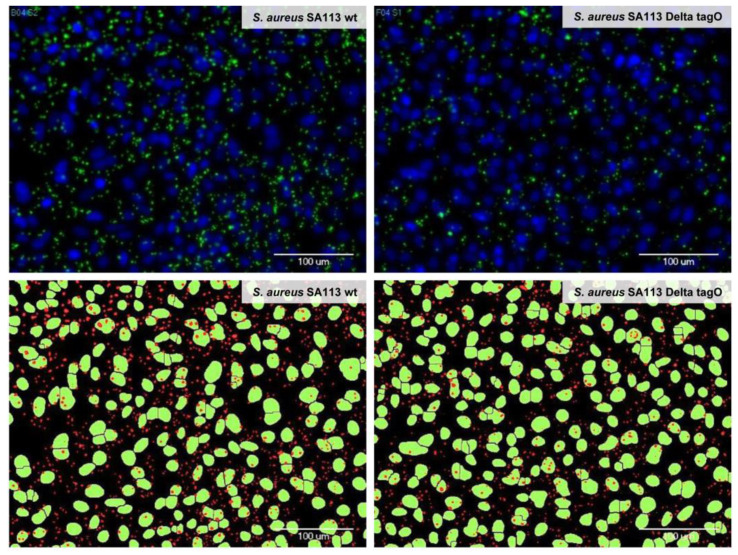
Identification of adherent bacteria by automated microscopy and image analysis. Adhesion of wild type *Staphylococcus aureus* SA113 (**left**) and *S. aureus* SA113 Delta tagO (**right**) to A-549 cells is depicted. Cell nuclei were stained with DAPI (**upper panel**, blue and **lower panel**, green) and bacteria were stained with a primary antibody against teichoic acid and an Alexa 488-labeled secondary antibody (**upper panel**, green and **lower panel**, red). Upper panel: Images acquired with the automated microscope after washing and staining; lower panel: Bacterial and epithelial cell areas as identified by the imaging software MetaXpress with the module “Transfluor” and the mode “Vesicle area per cell”, based on the microscopic images shown in the upper panel. A bacterial density of OD_600_ = 0.1 has been used (for quantification please refer to Figure S1).

The cell nuclei and the adherent bacteria were reliably identified by automated microscopy and image analysis. As illustrated in Figure 1, there was a good match between the microscopic pictures of the bacteria and the A-549 cells after adhesion, fixation, and fluorescence staining (upper pictures) and the results from the automated image analysis and the labeling of the recognized structures (lower pictures). By staining the cytoplasm of the A-549 cells in addition to the cell nuclei and the bacteria, we verified during the assay development that the bacteria did not migrate from their initial attachment site on the A-549 cells to sites on the well surface where the eukaryotic cells have been detached during the washing procedures (Figure S4). To conclude, the established automated microscope-based approach enabled the quantitative investigation of adhesion processes in microtiter plates and additionally revealed morphological changes that are induced to the cells upon exposure to the tested substances.

### 3.2. Screening of Compound Libraries for S. aureus Adhesion Inhibitors

After the optimization and validation of the assay conditions, bacterial adhesion was investigated in a screening approach with 4208 compounds (Table 1) at 20 µM concentration in duplicate. The compound collection consisted of six different parts. Our in-house collection of secondary metabolites from myxobacteria (part 1) is a unique source of highly bio-active compounds. Many of those compounds have shown antibacterial, antiherbal, cytotoxic, and other properties and are the focus of ongoing investigations by us and many others [27,29]. The LOPAC collection of pharmacologically active compounds (part 2) is a broad collection of a large number of drugs and drug-like molecules, most of which have already been studied in preclinical and clinical investigations. Hits that would be derived from the collection have the advantage that an approval as a drug can be aided by prior data that is already available, which may speed up the process considerably and reduce cost (“drug repurposing”). Our front-runner ATA was part of the LOPAC collection. The VAR collection (part 3) is also unique at our institute and provides a variety of unusual structures that are not found in most commercial substance collections and extended the “structure space” of our screening campaign considerably. The linear and cyclic peptides (part 4–6) are consisting of sublibraries with defined and randomized positions. Such libraries have been used successfully in other screening approaches by employing an iterative stepwise procedure to enhance biological activities. We considered this combination of unique and drug-like structures a solid basis for our project.

**Figure 2:**
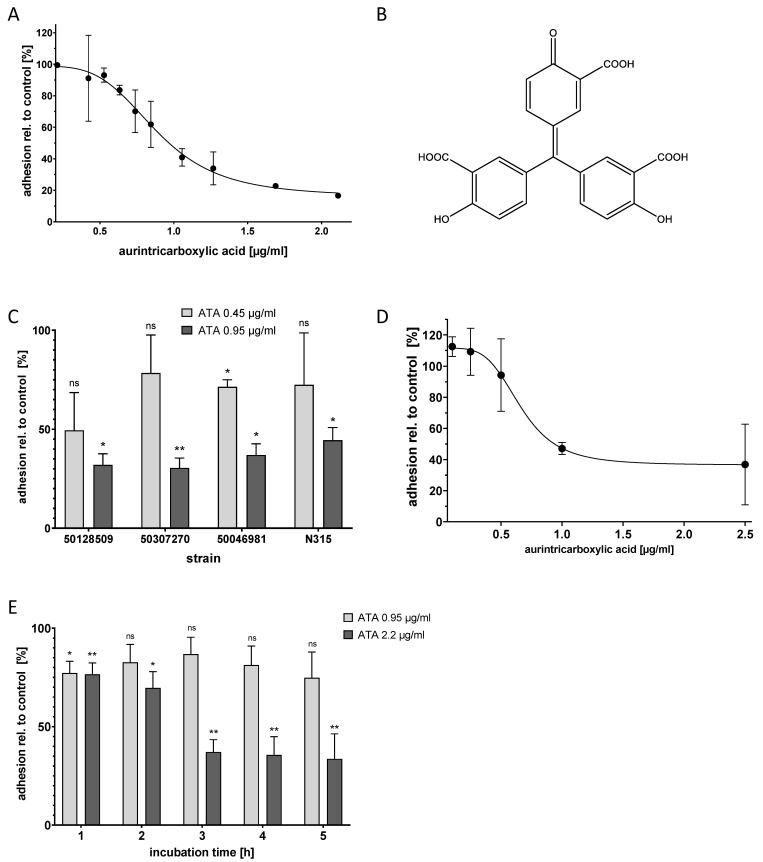
Aurintricarboxylic acid (ATA) inhibited *S. aureus* adhesion to A-549 cells and to human primary epithelial cells in a dose-dependent manner and reduced precolonization. (**A**) Adhesion of *S. aureus* N315 to A-549 cells was determined in the presence of different concentrations of ATA using the microscopic adhesion assay; Mean and standard deviation for three replicates are depicted. (**B**) Structure of ATA in its monomeric form. (**C**) ATA reduced the adhesion of *S. aureus* clinical isolates to A-549 cells. Mean and standard deviation for two replicates are depicted. * *p* < 0.4, ** *p* < 0.003, ns = not significant, as compared to control according to Welch’s unpaired *t*-test. (**D**) Adhesion of *S. aureus* to primary epithelial cells from human nares (HNEPC) in presence of ATA. Mean and standard deviation for three replicates are depicted. (**E**) Application of ATA to A-549 cells that were pre-colonized with *S. aureus* N315 resulted in the time-dependent detachment of the bacteria; incubation times are indicated. The values for each time point are related to controls without ATA. Mean and standard deviation for three replicates are depicted. * *p* < 0.014, ** *p* < 0.01, ns = not significant, as compared to control according to Welch’s unpaired *t*-test.

The adhesion inhibitor polyinosinic acid was used at 50 µg/mL as a positive control in each microtiter plate [36]. In the primary screen, 62 compounds were found to be active with at least 30% reduction of adhesion (Supplementary Tables S1 and S2). Substances that caused substantial loss of eukaryotic cells during the incubation were excluded from further testing. An advantage of using the automated microscope was the possibility to reinvestigate wells individually by visual inspection of the pictures that were recorded. Since the ratio of bacteria to cell nuclei was determined, the approach was, contrary to the microtiter plate readings, insensitive to partial losses of the epithelial cells during the washing procedures. The antimicrobial effect of the initial hits was evaluated in a broth dilution assay (MIC determinations). Substances with MIC values below 100 µM were excluded from further testing. After the re-evaluation process three compounds were identified that reliably and selectively reduced bacterial adhesion without detaching the eukaryotic cells and with MIC values > 100 µM (Table S3 and Figure S5; for structures see Figure 2B and Figure S6).

### 3.3. ATA Inhibited S. aureus Adhesion to Epithelial Cells In Vitro

The most active substance found in the screen was polyaromatic aurintricarboxylic acid (ATA, Figure 2B), which reproducibly reduced bacterial adhesion with a half-maximal inhibitory concentration (IC50) of 0.95 µg/mL. The maximum effect size was a reduction of adhesion to ca. 20% of the original value; this was reached at a compound concentration of approximately 2.2 µg/mL (Figure 2A).

Over the course of the adhesion experiment, ATA did not cause any apparent morphological effect on the A-549 cells. In addition, bacterial growth was not impaired by ATA up to a concentration of 42 µg/mL (100 µM, data not shown). In order to assess whether ATA exerts cytotoxic effects, an MTT test with A-549 cells was carried out. After 24 h of incubation, ATA caused less than half-maximum inhibition of metabolic activity of the A-549 cells up to the highest test concentration of 370 µg/mL (data not shown). Toxicities for ATA in mice have been reported before. A dose of 100 mg/kg/day per day i.p. was found to be lethal, whereas 30 mg/kg/day was not [37].

To analyze the activity of ATA towards different *S. aureus* strains, three clinical isolates of *S. aureus* were used, strain 50,307,270 (rifampicin resistant), strain 50,128,509 (MRSA), and strain 50,046,981 (MSSA). ATA could inhibit the adhesion of all strains with a potency that was equal to or higher than that found for N315 (Figure 2C).

Next, the effect of ATA on HNEPC was investigated. The results obtained with the immortalized epithelial cell line A-549 could be confirmed: The adhesion of *S. aureus* N315 to the primary cells was reduced with an IC50 of approximately 1 µg/mL (Figure 2D). Again, ATA did not completely block adhesion, but reduced it to approximately 40% of the original level.

In the primary screening assay, the compounds were already present when bacteria were added to the host cells. Thus, the assay mimics a preventive setting. In order to investigate whether ATA might also work in a therapeutic setting, its effect on already adherent bacteria was investigated next. For this purpose, A-549 cells were precolonized with *S. aureus* N315 for one hour, followed by the removal of non-adherent and planktonic bacteria by washing. Further incubations from one to five hours with 0.95 µg/mL and 2.2 µg/mL ATA (corresponding to the IC50 and maximal effective concentrations, respectively) were carried out. In comparison to the controls without an active compound, ATA was able to reduce the number of bacteria from precolonized A-549 cells in a dose- and time-dependent manner (Figure 2E). This demonstrates that ATA can disrupt an already existing bacterial adhesion, which suggests that ATA might be used in both preventive and therapeutic settings.

### 3.4. Drug Carriers Did Not Interfere with S. aureus Colonization

A suitable drug carrier has to distribute the active compound throughout the nasal cavity to facilitate *S. aureus* elimination [38], without affecting the bacterial load itself. In a pilot experiment, we compared the intranasal distribution of three drug carriers, Poloxamer 407, S/V, and PBS. We mixed them with Evans blue to visually inspect their distribution in the nasal cavity. Poloxamer 407 and S/V were chosen because of their very different viscosity and physical properties, which could influence their distribution in the nasal compartments. Whereas S/V is highly viscous at room temperature and needs to be warmed up to become liquid and pipettable, Poloxamer 407 is liquid at low temperature and turns into a gel at 37 °C. All substances were applied into the nasal cavity by inserting a Hamilton^©^ syringe with a shortened and blunted needle a few millimeters into the anterior nares. All three substances spread equally well throughout the entire nasal cavity 30 min after application. They were found in the ventral region close to the nares, along all nasal turbinates, and also in the most dorsal regions, such as the nasopharynx (data not shown).

**Figure 3:**
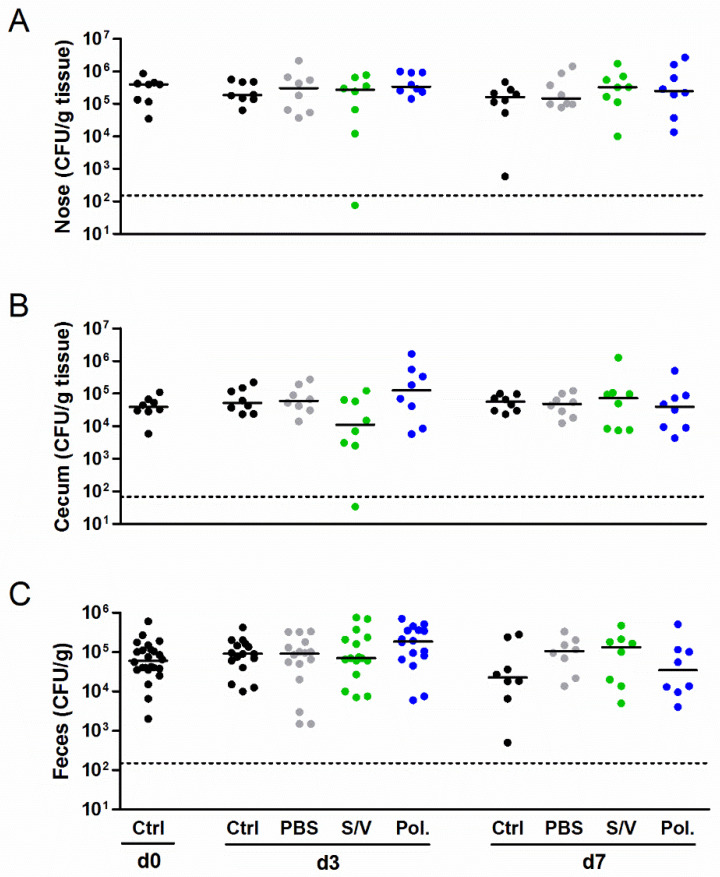
Drug carriers did not interfere with the bacterial burden in a persistent *S. aureus* colonization model. Female C57BL/6N mice were colonized intranasally with 0.7–1.0 × 10^8^ CFU *S. aureus* JSNZ. Starting on day 3 after colonization, mice were treated once daily for 7 consecutive days with 10 µL of the carrier substances PBS, Softisan 649/Vaseline, Poloxamer 407 or left untreated. The *S. aureus* burden was determined in the homogenized nose (**A**), cecum (**B**), and feces (**C**) 0, 3, and 7 days after starting the treatment. The detection limit is indicated by a dashed line; medians are indicated. Data were pooled from two independent experiments with 4 mice/group. Abbreviations: Ctrl—control; PBS—phosphate-buffered saline; Pol.—Poloxamer 407; S/V—Softisan 649/Vaseline 9:1 (*w*/*w*).

Next we studied the influence of the carrier substances on *S. aureus* colonization in vivo using a new mouse model of *S. aureus* nasal colonization/decolonization established by our group [21,22]. Mice were intranasally inoculated with the mouse-adapted *S. aureus* JSNZ strain to induce persistent colonization. Mice were colonized with JSNZ for three days and subsequently treated daily with Poloxamer, S/V or PBS without the active compound for 3 to 7 days. As expected, *S. aureus* JSNZ persistently colonized the nasopharynx and gastrointestinal tract of mice throughout the experiment (Figure 3). The tested drug carriers did not impact on *S. aureus* colonization, as reflected by constant bacterial loads in the nose, cecum, and feces. This advantageous result is in strong contrast to our preliminary findings in the cotton rat model (see below). A complete elimination of *S. aureus* in the nose and cecum was only observed in a single mouse treated for 3 days with S/V. These experiments demonstrated that all tested carrier substances are suitable for the delivery of candidate drugs in our *S. aureus* nasal colonization/decolonization model.

### 3.5. ATA Failed to Induce S. aureus Decolonization, While Mupirocin Was Highly Effective

**Figure 4:**
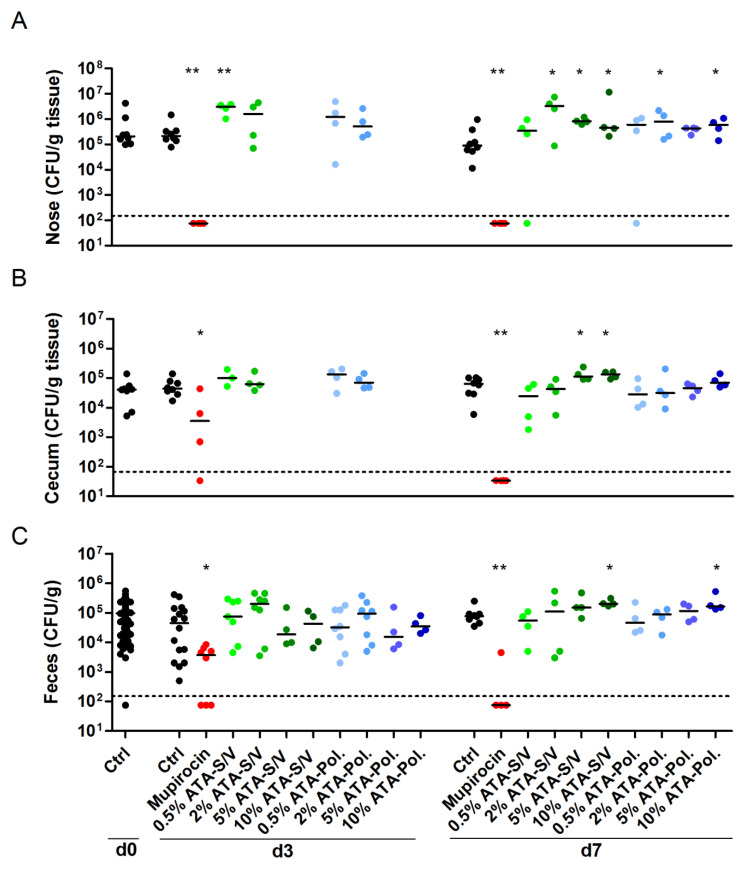
Mupirocin eradicated *S. aureus* colonization, while ATA was not effective. Female C57BL/6N mice were colonized intranasally with 0.7–1.0 × 10^8^ CFU *S. aureus* JSNZ. After three days of colonization, mice were treated on a daily basis for 7 days with 10 µL 2% Mupirocin or ATA in different concentrations (0.5%, 2.5%, 5% or 10%) using Softisan 649/Vaseline or Poloxamer 407 as drug carrier. Groups receiving 5% or 10% ATA were only analyzed at day 7. A control group remained untreated. The *S. aureus* bacterial load was determined in the homogenized nose (**A**), cecum (**B**), and feces (**C**) 0, 3, and 7 days after starting the treatment. The detection limit is indicated by a dashed line; medians are indicated. Data were pooled from two independent experiments with 4 mice/group. Statistics: Groups were compared with Ctrl using Mann-Whitney test, * *p* < 0.05, ** *p* < 0.01. Abbreviations: Ctrl—control; Pol.—Poloxamer 407; S/V—Softisan 649/Vaseline 9:1 (*w/w*).

Finally, we investigated whether the hit compound ATA reduces *S. aureus* nasal burden. We used Poloxamer and S/V as drug carriers. After three days of bacterial colonization, mice were treated for 7 consecutive days with ATA ointment in two different doses (0.5% and 2%). Its effect on the *S. aureus* load was compared with the human therapeutic agent mupirocin. As expected, the antibiotic mupirocin eradicated *S. aureus* from the nasal region even in a short treatment regime of 3 days (Figure 4A). Although applied only to the nasal cavity, mupirocin also eliminated *S. aureus* from the gastrointestinal tract of most mice within 7 days of treatment (Figure 4B,C). In contrast, ATA did not reduce the *S. aureus* burden in the nose, cecum or feces. This was true at concentrations of 0.5% (2.5 mg/kg body weight) or 2% (10 mg/kg body weight) and in combination with Poloxamer or S/V. Even very high ATA concentrations of up to 10% just reaffirmed the inefficacy of the compound in reducing *S. aureus* colonization. On the contrary, ATA treatment even increased the median nasal burden of *S. aureus* JSNZ (Figure 4). Moreover, mice treated with 10% ATA showed a slight reduction of weight when compared with untreated mice, resulting in a significantly higher disease activity index of this group (*p* = 0.0473 for 10% ATA-S/V; Figure S7). Altogether, the in vivo experiments demonstrate the robustness and suitability of our murine *S. aureus* nasal colonization/decolonization model for testing candidate drugs for *S. aureus* decolonization. ATA, however, failed to induce *S. aureus* decolonization.

## 4. Discussion

Due to increasing numbers of mupirocin-resistant *S. aureus*, new decolonization approaches are urgently required. In this study, we investigated pathoblockers, which means compounds that reduce the pathogenicity of the bacteria without interfering with their viability. Hence, adhesion inhibitors should exert less selection pressure than antibiotics and reduce the development of resistances. In accordance with this goal, we have sorted out compounds that showed antimicrobial effects (MIC values) below 100 µM in our further evaluation of the initial hits.

Our first task was the development of a method that allows the screening of several thousand substances for compounds that inhibit the adhesion of *S. aureus* to epithelial cells. For this purpose, an automated microscope was employed that allows the quantitative investigation of adhesion processes in microtiter plates and that can in addition be used to reveal morphological changes that are induced to the cells by the influence of the substances. Studies of the adhesion of bacteria to respiratory epithelial cells are frequently carried out with the human lung epithelial cell line A-549 [17,24,25,26,27,28,29,30,31,32], which was also employed in the present investigation.

In the human nose *S. aureus* is in a constant process of exponential growth due to ongoing excretion and shedding [39]. Such a situation suggests good chances for a therapeutic application of adhesion inhibitors. We report a medium throughput screening of more than 4.000 compounds for *S. aureus* adhesion inhibitors and the subsequent characterization of ATA, which was identified as the most potent compound in this assay. Our method works with unlabeled bacteria and eukaryotic cells during the adhesion process, which offers the possibility to use unmodified wild type bacterial strains and to capture any influences of the test compounds on the morphology of the eukaryotic cells in the initial screening campaign. This is an advantage over previously published procedures, as it allows the rapid exclusion of compounds that exert their effect mainly through toxic effects on the eukaryotic cells. It is likely that the approach for identifying adhesion inhibitors presented in this study can be applied to other settings, where the adhesion of pathogens to epithelial cells or eukaryotic cells in general is of interest. Bacterial adhesion to epithelial cells is the important first step in many clinically significant conditions, like the infection of the urinary tract with *Escherichia coli* [40], the colonization of the lungs with *Pseudomonas aeruginosa* [41] or the adherence of Streptococci to the pharynx [42]. By employing specific and adequate staining procedures, the approach described in this study can be adopted to the particular setting.

The number of bacteria that may have internalized into the A-549 cells has not been determined in our current protocol. Previous studies reported a low internalization rate with only 15% of A-549 cells being infected and low bacterial numbers per cell after 1.5 h of incubation [43]. Therefore, we do not expect a relevant influence of internalization events on our results.

For ATA several biological functions have been described [44,45,46,47,48,49,50], but the finding that ATA potently prevents the adhesion of *S. aureus* to epithelial cells was new. It should be noted that ATA, which is shown in its monomeric form in Figure 2B, has a strong tendency to oligomerize, which results in polyanionic structures [51]. We speculate that the activity of ATA to reduce adhesion of *S. aureus* to epithelial cells might be linked to its acidic functions, because several other polyanionic substances like polyinosinic acid and sulfated polysaccharides are known to inhibit the cellular adhesion of *S. aureus* [35,52,53,54]. Whereas the polypharmacological properties and the limited chemical stability render a systemic use of ATA challenging, a topical administration for the decolonization of the nose appeared attractive based on the cellular data.

Apart from the anti-adhesive effect reported here, ATA can act as a *S. aureus* pathoblocker by potent inhibition of Stp1 [55]. Stp1 is a Ser/Thr-phosphatase involved in the global regulation of staphylococcal virulence factors, such as alpha-hemolysin and leukocidins, immune-evasion molecules, such as SCIN and CHIPS, as well as capsular polysaccharide synthesis enzymes [56,57,58,59]. Hence, Stp1 inhibition switches *S. aureus* from an invasive, virulent state to a more silent, immune-evasive state. Administration of ATA significantly reduced the severity of staphylococcal infection in a murine abscess formation model [55].

ATA is also well-known as a small molecule inhibitor of nucleases and nucleic acid-binding enzymes [60]. Diverse studies have reported that ATA inhibits the replication of a variety of viruses in vitro by interference with viral polymerases and RNA-binding proteins [61,62,63]. Moreover, ATA compromised bacterial biofilm formation by limiting protein-nucleic acid interaction [64]. Whether this is also relevant to *S. aureus* biofilms, which are also composed of eDNA, remains to be investigated.

Despite being the top candidate in the in vitro screening approach, ATA was ineffective in promoting *S. aureus* decolonization in our mouse *S. aureus* colonization model, but even increased the bacterial burden when applied intranasally in a higher dose (10% ATA, equals 50 mg/kg body weight/day). We suggest that the anti-adhesive effect of ATA observed in our in vitro assay could have been masked in vivo by its anti-inflammatory activity [65,66,67,68]. It is well known that the innate and adaptive immune responses are critical for the clearance or persistence of *S. aureus* nasal colonization [69,70,71]. ATA, however, can inhibit innate and adaptive immune responses in many ways. The compound interferes with JAK-STAT signaling, thereby inhibiting IFN-ɣ-induced iNOS expression [68]. Moreover, ATA is able to block chemotaxis of dendritic cells and T cells [67], to inhibit complement activation in vitro [65], and to convert naïve CD4+ T cells into FoxP3+ regulatory T cells [66]. Collectively, these mechanisms could reduce the immune response against the colonizing staphylococci and thereby enhance bacterial persistence in the nasal cavity. In consequence, such an anti-inflammatory effect could override the anti-adhesive properties of ATA in the nasal colonization model. ATA’s anti-inflammatory effect could also explain other cases where a protective effect shown in vitro could not be reproduced in the living organism. In line with our data, intraperitoneal injection of ATA enhanced disease severity in murine models of vaccinia virus and orbivirus infection [44,72].

The in vivo evaluation of novel decolonization drugs requires a robust and persistent *S. aureus* colonization model with high discriminatory power between treated and untreated animals. In the past, researchers usually colonized laboratory mice with human-adapted clinical *S. aureus* isolates. Due to poor adaptation to the murine environment, human *S. aureus* isolates usually do not consistently colonize mice and are frequently eliminated from the murine nasal cavity within days [12,21,73,74]. Nevertheless, several drug candidates were investigated using this model [8,12]. However, the variable colonization rate and the rapid natural decolonization strongly reduced the signal-to-noise-ratio in these studies [12].

In search for a more robust and reliable animal model, Kokai-Kun et al. established a nasal colonization model in cotton rats [11]. In cotton rats there is consistent and persistent high-level (~5000 CFUs/nose) nasal colonization by *S. aureus* and consequently a high discriminatory power. This model has been used over the past two decades to evaluate antimicrobials, e.g., lysostaphin [11], cationic methacrylate polymers [13], epidermicin [75], and antimicrobial peptides [76]. Cotton rats are highly excitable rodents and hence much more difficult to handle than mice [77]. We carried out initial studies with ATA in the cotton rat model using human-adapted *S. aureus* strains. However, several drug carriers without drug led to a drastic decrease of the nasal bacterial load within just a few days, which made it impossible to discern any additional drug-related effect (data not shown). Hence, it is essential that the drug carrier distributes the active compound throughout the nasal cavity to facilitate *S. aureus* elimination [38], but does not affect the bacterial load itself. In our study, all three tested substances (Poloxamer 407, S/V, and PBS) spread equally well throughout the entire nasal cavity 30 min after application and did not impact on the *S. aureus* density in the nose.

We have recently reported that laboratory and wild mice are natural hosts of *S. aureus*, and that their colonizing strains show features of adaptation to their murine host. The prototype of these mouse-adapted strains, JSNZ, can colonize the murine nose and gastrointestinal tract for several weeks [21,59]. Colonization was induced in all inoculated animals with average nasal colonization loads even higher than in the cotton rat model (2 × 10^5^ CFU/g nose tissue, corresponding to 4 × 10^4^ CFU/nose). Our consistent and reproducible *S. aureus* colonization model now enables researchers to reliably evaluate novel decolonization drugs in the mouse.

The validity of our animal model is underlined by the results of mupirocin treatment, which served as benchmark for *S. aureus* decolonization. After three days of treatment nasal decolonization was 100%, decolonization of the gastrointestinal tract took a few days longer. In studies with mice that were colonized with human *S. aureus* strains or that used the cotton rat model, mupirocin frequently failed to completely decolonize the nose even at the clinically effective concentration of 2% mupirocin [8,12,13,76]. Apart from the animal model, this might also be due to differences in the experimental settings such as different application modes and schemes. Moreover, the employed carrier substances could have an impact on the spatial distribution of mupirocin within the nasal cavity and its release over time [11].

## 5. Conclusions

Adhesion inhibitors are a promising, but yet underexplored option to prevent or eliminate *S. aureus* colonization. The microscopy-based screening presented here is a powerful method to identify and test new *S. aureus* adhesion inhibitors. Our adhesion assay works with unlabeled bacteria and eukaryotic cells, which offers the possibility to use unmodified wild type bacterial strains and to capture any influences of the test compounds on the morphology of the eukaryotic cells in the initial screening campaign. Hence, it allows the rapid exclusion of compounds that exert their effect mainly through toxic effects on the eukaryotic cells. Importantly, this approach can be applied to other settings where the adhesion of pathogens to epithelial cells or eukaryotic cells in general is of interest.

The mouse-adapted strain JSNZ induces persistent nasal colonization and therefore provides an excellent tool for testing drug candidates against *S. aureus* nasal colonization in mice. In contrast to previous models using human-adapted *S. aureus* strains in mice, this model has a high discriminatory power between treated and untreated animals. While cotton rats can also be persistently colonized with *S. aureus*, their handling is much more difficult and less molecular tools are available to study the host response in detail. Even though our first candidate ATA failed to decolonize *S. aureus* from the murine nose, we are optimistic that our screening approach and persistent colonization model will provide a powerful tool for identifying effective antibacterial drugs in the future.

## Figures and Tables

**Table 1:** Substance collections for adhesion inhibitors screenings.

Type	Entities	Stock conc.
Secondary metabolites from myxobacteria ^1^	117	2 mM in DMSO
LOPAC collection of pharmacologically active compounds ^2^	1408	10 mM in DMSO
VAR collection ^3^	1600	5 mM in DMSO
Peptide library of the structure XXX12XXX-DKP made of D-amino acids ^4^	361	2.2 mM (in 2-propanol/water 1:1)
Peptide library of the structure XXX12XXX-DKP made of L-amino acids ^5^	361	4 mM (in 2-propanol/water 1:1)
Cyclic peptides of the structure [AA12AAC] made of D-amino acids ^6^	361	3.5 mM in DMSO
Total	4208	

^1^ Academic collection of the Helmholtz Centre for Infection Research (HZI), sourced from in-house myxobacterial research [27]. ^2^ Sigma-Aldrich/Merck (Darmstadt, Germany); LOPAC = abbreviation for ‘Library of Pharmacologically Active Compounds’. ^3^ Academic collection of the HZI, sourced from multiple medicinal chemistry groups; VAR stands for ‘various sources’. ^4^ X = mixture of all proteinogenic amino acids, 1 and 2 = defined amino acids (all proteinogenic amino acids in D-configuration), Cys excluded; DKP = diketopiperazine. ^5^ X = mixture of all proteinogenic amino acids, 1 and 2 = defined amino acids (all proteinogenic amino acids in L-configuration), Cys excluded; DKP = diketopiperazine; ^4,5^ prepared in the peptide synthesis facility of the HZI. ^6^ 1 and 2 = all proteinogenic amino acids in D-configuration, Cys excluded [28].

## Supplementary Materials

The following are available online at https://www.mdpi.com/2076-2607/9/3/631/s1, Figure S1: Adherence of the *S. aureus* SA113 Delta tagO mutant vs. the wild type strain, Figure S2: Adhesion of *S. aureus* N315 to A-549 cells after precultivation of the bacteria in BHI for different time periods Figure S3: Assessment of the performance of the adhesion test, Figure S4: Visualization of the adhesion of *S. aureus* N315 to A-549 cells, Figure S5: Reevaluation of the adhesion-reducing effect of the three most active compounds that were identified in the screening campaigns. Figure S6: Structures of the active compounds HZI10676D08 (methyl 7-hydroxy-9-methyl-6-oxo-6H-oxepino [2,3-b]chromene-5-carboxylate) and HZI10687B10 (pseudohypericin). Figure S7: Evaluation of the health status of the *S. aureus*-colonized mice after treatment with ATA or mupirocin.
