# Supplementary material for: Discovery of Staphylococcus aureus Adhesion Inhibitors by Automated Imaging and Their Characterization in a Mouse Model of Persistent Nasal Colonization
Source: Microorganisms. 2021 Mar 18;9(3):631. doi: 10.3390/microorganisms9030631 (PMC8002927; doi:10.3390/microorganisms9030631)
Supplement: Supplementary file 1 [file microorganisms-09-00631-s001.zip › microorganisms_1114982_supplementary figures_revision.docx]

**Discovery of *Staphylococcus aureus* adhesion inhibitors by automated imaging and their characterization in a mouse model of persistent nasal colonization**

Liliane Maria Fernandes de Oliveira, Marina Steindorff, Murthy N. Darisipudi, Daniel M. Mrochen, Patricia Trübe, Barbara M. Bröker, Mark Brönstrup, Werner Tegge, Silva Holtfreter

**Supplementary figures**

**Figure S1:** **Adherence of the *S. aureus* SA113 Delta tagO mutant vs. the wild type strain.** The adhesion of *S. aureus* SA113 and its isogenic mutant Delta tagO to A-549 cells was determined at different bacterial densities by an automated microscopy with image analysis. Mean and standard deviation of four replicates are depicted. *p < 0.04, **p < 0.01, ****p < 0.0001, according to Welch’s unpaired t-test.

**Figure S2: Adhesion of *S. aureus* N315 to A-549 cells after precultivation of the bacteria in BHI for different time periods.** Adhesion was determined with the automated microscope by quantification of the bacteria in relation to DAPI-stained cell nuclei according to the optimized procedure. Bacteria added at OD600 = 1.0. Orienting experiment with the quantification of 16 microscopic pictures per data point.

**Figure S3: Assessment of the performance of the adhesion test.** Confluent A-549 cells were incubated with *S. aureus* N315 in a 96-well microtiter plate followed by washing, fixation, fluorescence staining and analysis by automated microcopy and image analysis as well as by determination of fluorescence (485/535 nm) for the labelled bacteria in a microtiter plate reader. 92 wells without added compounds and four wells with 50 µg/ml polyinosinic acid (positive control) were used. The symbols without added compounds display the mean values of eight rows with 11 (4 rows) or 12 (4 rows) data points each. **** p < 0.0001 according to Mann-Whitney unpaired t-test.

~~
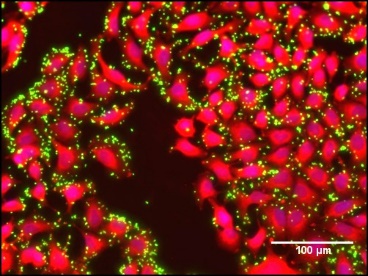
~~

**Figure S4:** **Visualization of the adhesion of *S. aureus* N315 to A-549 cells.** Staining of the cytoplasm with CellTracker™ Red CMPTX, cell nuclei with DAPI (blue) and bacteria with a primary antibody against teichoic acid and an Alexa 488-labelled secondary antibody (yellow). Fluorescence images aquired with the fluorescence filters “DAPI” for the detection of A-549 nuclei, “FITC” for the Alexa Fluor® 488 labelled bacteria and “Texas Red” for the imaging of cytoplasm. Shown are the superimposed images.

**Figure S5: Reevaluation of the adhesion-reducing effect of the three most active compounds that were identified in the screening campaigns at different concentrations.** Adhesion of *S. aureus* N315 to A-549 cells was determined in the presence of different concentrations of HZI10676D08 (methyl 7-hydroxy-9-methyl-6-oxo-6H-oxepino[2,3-*b*]chromene-5-carboxylate, HZI10687B10 (pseudohypericin) and aurintricarboxylic acid (ATA) using the microscopic adhesion assay. Orienting experiment with single well assays. Mean and standard deviation for nine areas that were analyzed in each well are depicted.



A B

**Figure S6:** Structures of the active compounds HZI10676D08 (methyl 7-hydroxy-9-methyl-6-oxo-6H-oxepino[2,3-*b*]chromene-5-carboxylate, **A**) and HZI10687B10 (pseudohypericin, B).

**
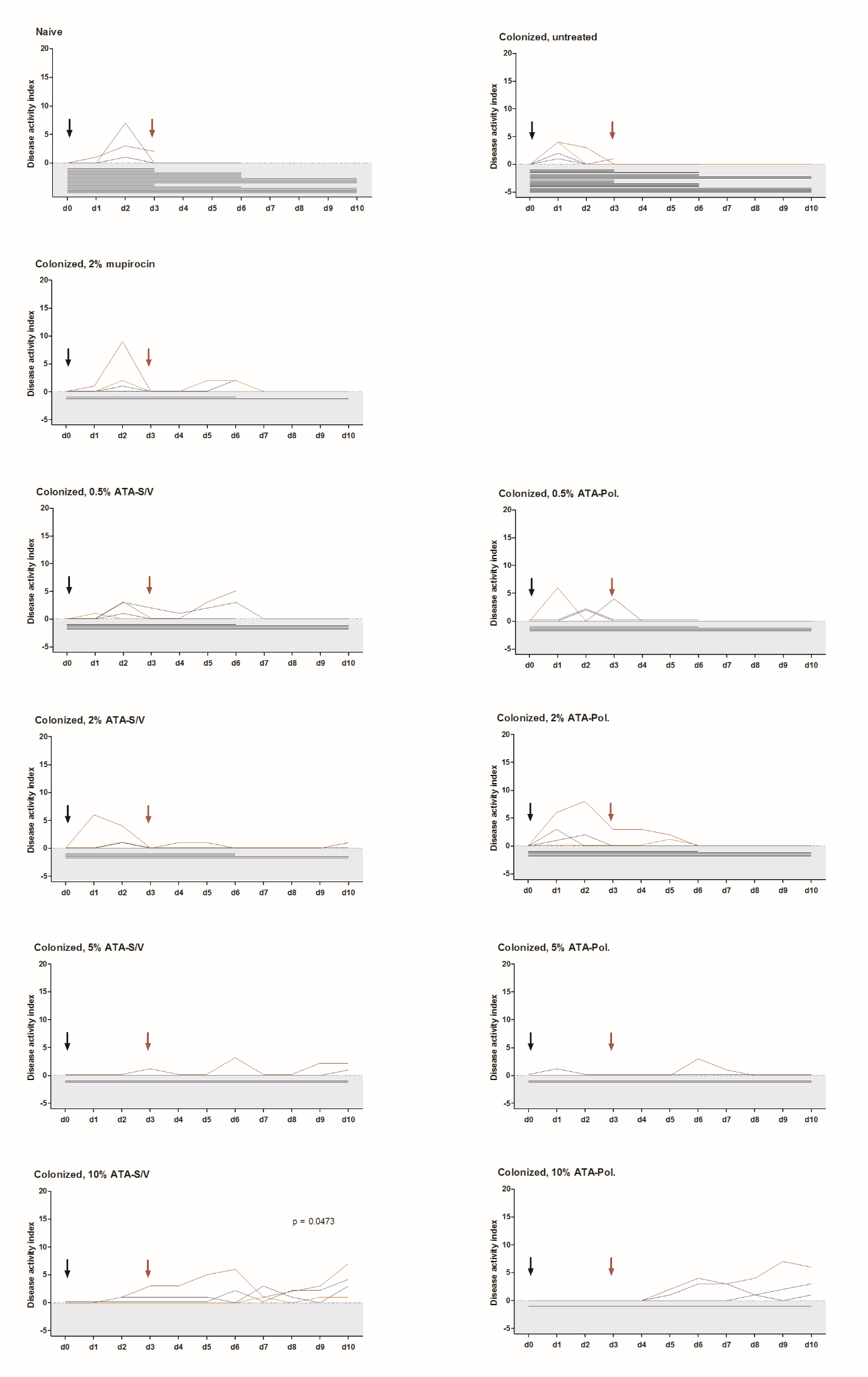
Figure S7:** **Evaluation of the health status of the *S. aureus*-colonized mice after treatment with ATA or mupirocin.** Female C57BL/6N mice were colonized intranasally with 0.7 – 1.0 x 10^8^ CFU *S. aureus* JSNZ (black arrow). After three days of colonization, mice were treated on a daily basis for 7 days with 10 µL 2% Mupirocin or ATA in different concentrations (0.5%, 2.5%, 5% or 10%) using Softisan 649 / Vaseline or Poloxamer 407 as drug carrier (red arrow). A control group remained untreated. The health status of all mice was scored on a daily basis after bacterial inoculation. The score comprises changes in weight, eyes and fur appearance, spontaneous and induced behavior, breathing, motility, posture, and faeces texture, and ranged from 0 to 20. Scores that remained zero throughout the study are displayed in the grey box. Statistics: Scores after treatment (d7-d10) were compared to the d0 score using Kruskal-Wallis test and Dunn’s multiple comparisons test, p value is indicated in the graph. Abbreviations: Pol. – Poloxamer 407; S/V - Softisan 649 / Vaseline 9:1 (w/w).
