# Supplementary material for: Discovery of Staphylococcus aureus Adhesion Inhibitors by Automated Imaging and Their Characterization in a Mouse Model of Persistent Nasal Colonization
Source: Microorganisms. 2021 Mar 18;9(3):631. doi: 10.3390/microorganisms9030631 (PMC8002927; doi:10.3390/microorganisms9030631)
Supplement: Supplementary file 1 [file microorganisms-09-00631-s001.zip › microorganisms_1114982_Supplementary Tables1-3_revision.docx]

Discovery of *Staphylococcus aureus* adhesion inhibitors by automated imaging and their characterization in a mouse model of persistent nasal colonization

Liliane Maria Fernandes de Oliveira, Marina Steindorff, Murthy N. Darisipudi, Daniel M. Mrochen, Patricia Trübe, Barbara M. Bröker, Mark Brönstrup, Werner Tegge, Silva Holtfreter

**Supplementary tables**

**Table S1: Number of active compounds found in the initial screening campaigns**

| **Type** | **Entities** | **No of initial hits** | **% of collection** |
| --- | --- | --- | --- |
| Secondary metabolites from myxobacteria | 117 | 0 | - |
| LOPAC collection of pharmacologically active compounds | 1,408 | 15 | 1.1 |
| VAR collection | 1,600 | 45 | 2.8 |
| Peptide library of the structure XXX12XXX-DKP made of D-amino acids | 361 | 0 | - |
| Peptide library of the structure XXX12XXX-DKP made of L-amino acids | 361 | 0 | - |
| Cyclic peptides of the structure [AA12AAC] made of D-amino acids | 361 | 2 | 0.6 |
| Total | 4,208 | 62 |  |

**Table S2: List of the most active compounds found in the initial screening campaigns**

| **Substance collection** | **Active compound** |
| --- | --- |
| LOPAC collection of pharmacologically active compounds (10 most active compounds) | Aurintricarboxylic acid (ATA) |
|  | Cantharidin |
|  | Cantharidic Acid |
|  | 7-Cyclopentyl-5-(4-phenoxy)phenyl-7H-pyrrolo[2,3-d]pyrimidin-4-ylamine |
|  | 5-Hydroxyindolacetic acid |
|  | MDL 28170 |
|  | (-)-MK-801 Hydrogen maleate |
|  | Phorbol 12-myristate 13-acetate |
|  | Quinacrine dihydrochloride |
|  | U-75302 |
|  |  |
| VAR collection (20 most active compounds) | HZI10672F03 |
|  | HZI10672H07 |
|  | HZI10673B05 |
|  | HZI10673D05 |
|  | HZI10673F01 |
|  | HZI10674D05 |
|  | HZI10674H06 |
|  | HZI10676A01 |
|  | HZI10676A09 |
|  | HZI10676D08 |
|  | HZI10676E05 |
|  | HZI10685H11 |
|  | HZI10686B06 |
|  | HZI10686B11 |
|  | HZI10686C02 |
|  | HZI10686D09 |
|  | HZI10686E06 |
|  | HZI10686H09 |
|  | HZI10687B10 |
|  | HZI10687G02 |
|  |  |
| Cyclic peptides of the structure [AA12AAC] made of D-amino acids (two most actives) | [A-A-R-R-A-A-C(SH)]_cyclic_ |
|  | [A-A-R-K-A-A-C(SH)]_cyclic_ |

**Table S3: The three most active compounds after the reevaluation process**

| Compound | IC50 [µg/ml]* |
| --- | --- |
| aurintricarboxylic acid (ATA)** | 0.95 |
| HZI10676D08*** | 15 |
| HZI10687B10**** | 26 |

*approximate values; ** member of the LOPAC collection; ***/**** members of the VAR collection;

**** = pseudohypericin; for structures of ATA see Figure 2B and for HZI10676D08 and HZI10687B10 see Figure S6.
